# A Crisis and Its Solution

**Published:** 1919-01-11

**Authors:** 


### THE PREVENTION OF VENEREAL DISEASE.

### A CRISIS AND ITS SOLUTION.

In the *Times* of 4th inst. a letter was published, signed "Not a Medical Man," which replied to the objection to prophylaxis on the part of Lord Sydenham, Sir Thomas Barlow, and some clergy and dissenting ministers on behalf of the National Council for Combating Venereal Diseases, on the ground that it is immoral.

On December 28 Sir William Osler, Regius Professor of Medicine, Oxford, and some of the most distinguished members of the profession, in a letter to the *Times* wrote: "In view of this daily increasing menace to the health of the nation, is it not time that the present timid and hesitating counsels, leading as they must do to weak and ineffective action, should give place to clear advice and strong purpose? Venereal diseases, like other infectious diseases due to micro-organisms, can be successfully conquered only by careful study of their natural history and causation, and by the translation of that knowledge into action. Extraneous considerations can have no place in sanitary problems; it is because they have had too much place that the National Council has failed. Smallpox and typhoid have been largely banished by the adoption of methods based on knowledge of these diseases. Venereal diseases should be similarly dealt with. It has been abundantly proved during the war that venereal diseases can be controlled by the adoption of simple sanitary measures, the success of which is striking, and the materials for which can be obtained from any chemist. These measures should at once be made known and available, and an organised instruction given in their application. This is the only effective way of meeting a dangerous situation—dangerous not only for our soldiers and sailors, but for the population at large. The danger of delay is very great."

The discussion thus raised in the *Times* is of vital importance to the health and well-being of all classes throughout the nation. It is the clear duty of the Press, and especially of the medical Press, to make the position and its dangers clearly understood by people of all classes. We have therefore decided to publish the *Times* leading article on "The Prevention of Syphilis," which sets out the position ably and clearly, together with the layman's letter.

### The Prevention of Syphilis.

"Many diseases are more immediately fatal to mankind than syphilis, but none is so disastrous in its effects. To take but two examples of its destructive incidence, it is known that to it more than half of both the blindness and the lunacy in this country is directly due. Until recently there was no certain cure and no certain means of prevention. Medical science has now provided us with both; and, if advantage is taken of its discoveries, there is a prospect that within a few years one of the most appalling scourges of modern life will be almost unknown. In these circumstances it would be mere cowardice to allow a natural distaste for handling an unsavoury subject to stand in the way of the public interest because its details can be discussed only in the medical Press or in the consulting-room. It was clearly this view which led Sir William Osler, Regius Professor of Medicine in the University of Oxford, and some of the most distinguished members of his profession, to make a plain statement of the facts in our columns on December 28. Three days later Lord Sydenham, on behalf of the National Council for Combating Venereal Diseases, joined issue with the signatories as to the policy to be pursued in making the discovery known. There is, it is important to understand, no difference of opinion as to its effectiveness. Since the publication of these rival views we have received, however, a large number of letters from correspondents, from which it is clear that the public does not understand the point at issue. This is quite simple, and we propose to state it. Syphilis can be prevented by the use of calomel before infection, and less certainly by its use within an hour or two after infection. The disease does not develop for some days after infection. Sir William Osler and his co-signatories advocate the former method, and the National Council the latter. Medical evidence indisputably supports the former. There remains the moral argument. It used to be said that syphilis is a judgment for sin, but that argument has not been used since the nature of congenital syphilis was understood. 'The fathers have eaten sour grapes, and the children's teeth are set on edge.' Opponents of prevention have now shifted their ground, and argue that the fear of syphilis is an effective moral agent. Hospital statistics do not confirm this view; and, if they did, civilisation has surely outgrown the belief that fear can be the true motive of right conduct. The country owes much, and will owe more, to the educational campaign of the National Council; for in the abolition of venereal disease the exponent of right conduct, be he teacher or priest or lay reformer, must necessarily play a leading part; but, owing to its hereditary nature, he will never succeed if he refuses the help which medical science can now give. If these arguments are valid, as we believe them to be, a simple amendment of the Venereal Diseases Act, 1917, is necessary, in order that the preventive medicine may be easily obtained under the necessary safeguards."—The *Times* Leading Article.

### A Layman's View.

Sir,—The time has come for a little plain speaking. It is admitted by doctors that if certain simple precautions are taken before, or within a short time after, the act, the danger of infection is practically nil. Treatment before the act is termed "prophylaxis"—afterwards "early treatment." Prophylaxis is objected to by Lord Sydenham, Sir Thomas Barlow, and some clergy and dissenting ministers on the ground that it is immoral, but with a few exceptions they acquiesce in early treatment. If the critical hour is, say, 11 p.m., they object to the use of disinfectants at 10.45, but raise no objection if the treatment is used at 11.15. The sinner must not prepare for immorality, but he may use the best means known to science to escape the consequences. How this attitude of mind is justified I have not yet been able to discover, although I can appreciate the position of those who say that the Almighty has sent venereal disease as a punishment for immorality, and that no steps are justified to interfere with this dispensation.

But even these stalwarts are not prepared to go the length of denying medical treatment to those who are suffering from the disease. Indeed, everyone agrees that the infected person should submit to treatment at the earliest possible moment, so as to minimise the attack and the danger to himself and others. Of course, the whole subject bristles with difficulties. No decent person wishes to make vice easy. On the other hand, for the time being we must take mankind as they are. We must do our best to prevent the health of our splendid young men from being ruined by a preventible disease. Further, we must remember that the sinner is not the only person concerned. He may communicate the disease to others, and he may communicate it to his children. The attention of the public should not be diverted by a controversy as to the respective merits of prophylaxis and early treatment.

The point is that the disease can be prevented by the use of certain simple disinfectants, the names of which should be made widely known. Have the National Society for the Prevention of Venereal Disease the courage to give the public the names of the disinfectants? Not being a medical man, I do not feel disposed to take the responsibility. Huge sums are being spent in treating the disease. Why not let the people know how it can be stopped?—I am, Sir, your obedient servant,

January 1. Not a Medical Man.

